# Quality of Life in CKD Patients on Low-Protein Diets in a Multiple-Choice Diet System. Comparison between a French and an Italian Experience

**DOI:** 10.3390/nu13041354

**Published:** 2021-04-18

**Authors:** Antioco Fois, Massimo Torreggiani, Tiziana Trabace, Antoine Chatrenet, Elisa Longhitano, Béatrice Mazé, Francoise Lippi, Jerome Vigreux, Coralie Beaumont, Maria Rita Moio, Giorgina Barbara Piccoli

**Affiliations:** 1 Nèphrologie et Dialyse, Centre Hospitalier Le Mans, 194 Avenue Rubillard, 72037 Le Mans, France; afois@ch-lemans.fr (A.F.); maxtorreggiani@hotmail.com (M.T.); tizi.trb87@gmail.com (T.T.); achatrenet@ch-lemans.fr (A.C.); bmaze@ch-lemans.fr (B.M.); flippi@ch-lemans.fr (F.L.); jvigreux@ch-lemans.fr (J.V.); cbeaumont@ch-lemans.fr (C.B.); mariaritamoio@gmail.com (M.R.M.); 2 Department of Clinical and Experimental Medicine, Unit of Nephrology and Dialysis, A.O.U. “G. Martino”, University of Messina, 98124 Messina, Italy; elisa.longhitano@libero.it

**Keywords:** low-protein diet, elderly, CKD, chronic kidney disease

## Abstract

Prescribing a low-protein diet (LPD) is part of the standard management of patients in advanced stages of chronic kidney disease (CKD). However, studies on the quality of life (QoL) of patients on LPDs are lacking, and the impact these diets have on their QoL is often given as a reason for not prescribing one. We, therefore, decided to assess the QoL in a cohort of CKD stage 3–5 patients followed up by a multiple-choice diet approach in an outpatient nephrology clinic in France. To do so, we used the short version of the World Health Organization’s quality of life questionnaire and compared the results with a historical cohort of Italian patients. We enrolled 153 patients, managed with tailored protein restriction in Le Mans, and compared them with 128 patients on similar diets who had been followed in Turin (Italy). We found there were no significant differences in terms of age (median 73 vs. 74 years, respectively), gender, CKD stage, and comorbidities (Charlson’s Comorbidity Index 7 vs. 6). French patients displayed a greater body mass index (29.0 vs. 25.4, *p* < 0.001) and prevalence of obesity (41.2 vs. 15.0%, *p* < 0.001). Baseline protein intake was over the target in France (1.2 g/kg of real body weight/day). In both cohorts, the burden of comorbidities was associated with poorer physical health perception while kidney function was inversely correlated to satisfaction with social life, independently of the type of diet. Our study suggests that the type of LPD they follow does not influence QoL in CKD patients and that a personalized approach towards protein restriction is feasible, even in elderly patients.

## 1. Introduction

The central importance of low-protein diets (LPD) in the management of chronic kidney disease (CKD) was recently underlined in the new KDOQI guidelines on nutrition in kidney diseases, which highlight the advantages of protein restriction and broaden the perimeter of action of these diets, suggesting that low-and very low-protein diets (LPD and vLPD), respectively, defined as supplying 0.6 g (moderate protein restriction) or 0.3–0.4 g of proteins per kg of ideal body weight per day, may be indicated as early as CKD stage 3 [1].

While these positions reinforce the enthusiasm of teams that have experience in using dietary management for patients with CKD, perplexities about their feasibility persist. Frequently, three points are raised: the risk of malnutrition, the difficulties encountered in obtaining compliance, and the risk of affecting a quality of life that is already threatened by a chronic disease [2,3].

Following several large trials and observational studies it has been realized that the risk of impairing nutritional status is much lower than was once thought and it is now held that, when correctly prescribed and followed, these diets do not lead to protein-energy wasting, and may even result in an improvement in nutritional status, in spite of protein reduction [4,5,6,7,8]. There are several reasons for this: focusing attention on energy intake may lead to the optimization of protein metabolism; a lower protein intake, in particular of proteins of animal origin, often leads to better control of acidosis and improves the calcium–phosphate balance; and attention to quality of food can limit exposure to toxic additives, including, but not limited to, inorganic phosphate [4,5,6,7].

The compliance issue is of pivotal importance. The findings have differed in trials, in which dietary treatments are usually standardized and patients cannot choose their own dietary regimen, and in observational studies, in particular when these studies were performed in settings in which patients’ preferences were taken into account in defining their diet strategy [9,10,11,12]. In such settings, the model of care shifts from compliance to concordance, a term that suggests mediating between patients’ wishes and preferences and physicians’ best options, reaching a feasible compromise adapted to each individual [13].

Patient motivation and the presence of a patient-friendly system of care can help to optimize the effect of dietary prescriptions. Several strategies have been proposed, including educational sessions, strict follow-up, the inclusion of unrestricted meals, and the possibility of periodically changing dietary approaches to reduce diet tiredness [14].

In addition to the classic indication of CKD stages 3–5 not on dialysis, some new indications for moderately protein-restricted diets are emerging, including renewed interest in using them for incremental dialysis and high-risk pregnancies [15,16,17]. Furthermore, low-protein diets are occasionally employed as a rescue treatment for patients with severe proteinuria who were found to be unresponsive to specific treatment or to conventional supportive management with angiotensin-converting enzyme inhibitors or angiotensin receptor blockers [18,19,20,21]. Conversely, normalization of protein intake (0.8 g/kg/day) is occasionally offered to patients with a very high protein intake or with a slow progression of kidney disease and advanced age [14].

In this broad, heterogeneous context, we lack studies specifically addressed to the assessment of the quality of life in CKD patients on protein-restricted diets. Although this knowledge gap was underlined in the recent Cochrane review of diet in CKD, since its publication only one large study from Italy on this question has been carried out [22,23,24].

In this context, we will describe the results obtained in a large French CKD cohort, encompassing mainly elderly and high-comorbidity patients. Quality of Life (QoL) was assessed in the context of an observational prospective study whose aim was to evaluate the implementation potential and the long-term impact of LPDs in the management of advanced CKD. In the setting of the study, LPDs are proposed in a flexible way, with a step-wise reduction in protein intake and are adapted to patients’ preferences. The results were compared with a historical cohort of on-diet patients in Italy, followed in a setting employing the same philosophy of flexibility and adapted care [10].

## 2. Materials and Methods

### 2.1. Study Setting

This study was conducted in France, at the Centre Hospitalier le Mans (CHM), in the unit for the care of advanced kidney disease (UIRAV—(nité pour l’Insuffisance Rénale chronique AVancée) [14]. The CHM is one of the largest non-university hospitals in France, with about 1750 beds (20 for nephrology), whose nephrology unit offers care from the initial stages of kidney disease to dialysis and follow-up after transplantation (which is performed by neighboring university hospitals).

Overall, two senior nephrologists, three dieticians, one resident, and a small group of nurses work in the UIRAV. Psychological care is provided by a dedicated psychologist. Patients are followed up with outpatient visits or in day-hospital if they need intravenous treatments or complex diagnostic assessments, including at least four consultations or imaging investigations. Conventional hospitalization in the nephrology ward is available if needed.

The UIRAV follows patients in CKD stages 4 and 5 or with progressive stage 3, up to the start of dialysis or transplantation. Whenever possible, dialysis is started with an incremental policy. Home hemodialysis and peritoneal dialysis trainings are also performed in this unit.

The results of this study are compared to those recorded in a previous study (TOPI) in a center located in Turin in Northern Italy [23]. This was a small university center with three nephrologists, and one part-time fellow dietician; its day hospital performed about 2500 hospitalizations per year.

Both the Turin and the Le Mans units were set up by the same senior nephrologist, but their socio-cultural context differs in many ways. The city of Le Mans is located in a rural area in central France, where most people have a high baseline protein intake. Conversely, in Turin, as overall in Italy, traditional Mediterranean habits favor low-protein diets. Moreover, in Italy protein-free products are available and fully reimbursed while in France they are not.

In spite of different baseline habits, the same diet policy was adopted in both settings, as patients in the Turin unit were followed up by the same senior nephrologist who later organized the unit in Le Mans.

### 2.2. Diet Options and General Policies

The common goal in both settings was to employ a moderately protein-restricted diet (LPD, 0.6 g/kg/day) in patients with progressive CKD stage 3, or those in stages 4 or 5 not on dialysis, in the absence of signs of protein energy wasting (PEW), alimentary disorders, or very short life expectancy (less than 3 months). This goal is usually attained in several steps, of which normalization of the protein intake is usually the first, in particular in cases with high baseline protein intake; very old patients and patients with a slowly progressive kidney disease may be managed with a normalized diet, while motivated patients, able to follow a 0.6 g/kg/day diet may attempt to further restrict their protein intake and follow a supplemented very LPD (0.3–0.4 g/kg/day).

As a first step, a dietician assesses the baseline protein intake and a nephrologist prescribes normalization or restriction of the protein intake; the prescription is individualized and is based on the patient’s baseline habits and nutritional status, and the trajectory of their CKD progression, also considering proteinuria, age, comorbidity, and life expectancy.

The assessment of protein intake at baseline and over follow-up is usually based on a dietary journal reviewed by the dieticians, as discussed below.

The clinical suggestions are discussed with the patient and the main type of protein intake (mixed proteins or plant-based) is agreed on. Since, in most cases, the baseline dietary protein intake is high, a stepwise approach “French style”, from normalization to restriction, described in greater detail in a previous study, is usually followed [14].

Most diets are: “traditional”, based on the usual dietary patterns in the area. They usually include one vegetarian meal per day, in general a vegetable soup, containing potatoes as a source of starch, accompanied by small portions of dairy products, in keeping with traditional meals in rural France, while meat or fish, combined with bread, rice, pasta, or potatoes, are the main course in the other meal. Conversely, plant-based diets rely on carbohydrates such as potatoes, rice, bread, and pasta as their main sources of calories, and favor proteins of vegetable origin (from grains and beans). Fruits are limited only in diabetic patients; overall there are no restrictions on vegetables.

Patients are followed up to identify signs of PEW, such as reduction in body weight (unexplained by edema reduction), reduction in lean body mass (evaluated by clinical assessment integrated with bioimpedance when deemed necessary), reduction in serum albumin, prealbumin, or total proteins, in the absence of acute inflammatory events, or other clinical markers of poor nutrition, in the presence of vitamin deficits or unexplained anemia.

The LPD may require the supplementation with a mixture of amino acids and ketoacids (Kestosteril, which is available free for CKD patients in both France and Italy).

The daily dose, for moderately restricted diets, in keeping with previous experience, is 1 tablet per 8–10 kg of body weight, and can be further adjusted on the basis of albumin levels or protein losses [14]. Occasionally, diets with normal protein intake (i.e., 0.8 g/kg/day) require keto-analogue supplementation. This may be due to signs of malnutrition, or in case of nephrotic proteinuria.

Protein intake is assessed per kilogram of real body weight, and an average between real and ideal body weight is used only for patients whose body mass index (BMI) is >40 kg/m^2^.

Energy intake is tailored to 30–35 kcal/kg of body weight per day in non-obese younger patients; a total of 20–25 kcal/kg of body weight per day is considered acceptable for very old patients (>80 years of age) or for obese patients. Whenever possible, obese patients are urged to increase physical activity. The start of dialysis is decided within an “intent to delay” policy based on the usual clinical and biochemical markers of blood pressure control, fluid overload, hyperparathyroidism, or any clinical element suggesting uremic toxicity (anorexia, weight loss, nausea, malnutrition, restless leg syndrome).

The Le Mans center widely employs an incremental dialysis policy; however, in this study only patients not on dialysis were enrolled, in analogy with what had been done in Turin.

The management of sodium, potassium, phosphate, bicarbonate, folic acid, iron, erythropoietin, vitamin D, and vitamin B12, is tailored to blood levels and follows the usual rules of good clinical practice [1].

### 2.3. Study Design, Patient Selection, Inclusion Criteria, and Long-Term Follow-Up

Participation in the study was offered to all adults (>18 years of age), with a baseline estimated glomerular filtration rate (eGFR) of <60 mL/min/1.73 m^2^ (assessed by the CKD-EPI equation) [25]. Pregnant women, patients who refused to allow anonymous clinical data collection, and patients unable to give their consent were excluded from the study.

Patients were recruited from April 2018 to July 2019. Follow-up continued until December 2020 (data were censored according to mortality and dialysis start).

In Turin, the analysis took place from July to December 2014 and follow-up was concluded in February 2016.

### 2.4. Assessment of QoL

Quality of Life (QoL) was assessed by means of the short version of the World Health Organization’s quality of life questionnaire (WHOQOL-BREF) validated for CKD patients. Patients could choose whether to complete the questionnaires at home or fill them in while waiting for their clinical visit, and in selected cases, with the help of the nephrologist or resident.

The WHOQOL-BREF, which comprises 26 items, measures four different domains (physical health, psychological health, social relationships, and environment), was analyzed per question and per domain. The analysis was compiled using the standard indications for the questionnaire: scores 1 and 2 (lower scores) and scores 4 and 5 (higher scores) were analyzed together.

### 2.5. Data Gathered

The following data were gathered: demographic (gender, age, country of origin), type of kidney disease, as defined by the clinical charts; type of diet (previous diets, diet at the cross-sectional analysis, at each change of type of diet and outcome on dialysis or transplantation or death at last follow-up).

Comorbidity was assessed using the Charlson Comorbidity Index (CCI, scale: 0–33) [26]. The patient’s nutritional status was assessed by means of the Malnutrition-Inflammation Score (MIS, scale: 0–30) and the Subjective Global Assessment (SGA: A, B or C) [1,27,28].

Clinical data included height, weight, body mass index (BMI), and blood pressure; laboratory data included urea, creatinine, electrolytes, albumin, total serum proteins, hemoglobin and parathyroid hormone. Data not shown in the tables, but recorded in the database, are available on request.

Energy and protein intake were assessed by a dietician using the patient’s 7-day food diary or, in its absence (non-adherence, older age, etc.) based on the patient’s dietary recall. Analysis of 24 h urinary urea was employed for assessment of protein intake, employing the Maroni–Mitch formula in patients able to correctly perform a 24 h urine collection [29]. However, in this population of mainly elderly patients, their dietary journal was the preferred means of assessment. In the case of a discrepancy between the two measures, the results were discussed with the senior nephrologist, and the most reliable measure was retained.

Estimated glomerular filtration rate (eGFR) was assessed using the MDRD short and the CKD Epidemiology Collaboration (CKD-EPI) formulas [25,30]. Due to its wider use, the latter was employed in the study.

### 2.6. Statistical Analysis

Statistical analyses were performed using SPSS Statistics version 23 (IBM Corp., Armonk, NY, USA). Quantitative data were expressed as medians (min–max) and qualitative data were presented as proportions and percentages.

The normality and homoscedasticity hypotheses were tested with the Shapiro–Wilk and Levene’s tests, respectively, for continuous series. In case of normal distribution, Student’s *t*-test was performed to compare two groups (e.g., Le Mans vs. Turin) and variance analysis was performed for comparisons between three or more groups; otherwise, the Wilcoxon Rank Sum Test or the Kruskal–Wallis Test were chosen.

Multiple linear regression analysis was performed employing the QoL scores for each domain as outcomes, and testing the main clinical parameters including age (dichotomized at 70), gender, eGFR (dichotomized at 15 mL/min/1.73 m^2^), and CCI (dichotomized at 7).

Proportions were tested using the Chi-square test or Fisher’s exact test in case of low sample size. A two-sided alpha risk was set at 5%.

### 2.7. Ethical Issues

The study was conducted in accordance with the Declaration of Helsinki.

In Le Mans, the study was registered with the name of Pro-Re Re-Pro ((PROtéger les REins avec un REgime bas en PROtéines): protecting the kidneys with a low protein diet), and was approved by the Ethical Committee of the University Hospital de Toulouse (Comité de Protection des Personnes (CPP)) on 7 December 2018.

The patients in the control group from Turin were enrolled in a study called PROTERENE (ridurre le PROTEine per PROTEggere il RENE): reducing proteins to protect the kidney) that was approved by the Ethics Committee of the University of Turin, Italy (*delibera* 282, 28 January 2015).

Informed consent was obtained for anonymous management of clinical data from each patient at the start of follow-up in each center. Further consent for publication was not needed for this study, dealing with overall data, not with individual cases.

## 3. Results

### 3.1. Baseline Data

The study population consisted of 153 patients referred to the UIRAV unit.

Table 1 reports the main characteristics of the population, compared to the cohort described in the Turin study (*n*: 128). There was no difference in the male–female distribution. In both cohorts, the population was elderly, with a median age of 73 in Le Mans, and 74 in Turin. Comorbidity, evaluated by the Charlson Comorbidity Index (CCI) was likewise relatively high, with a median of 7 in Le Mans and 6 in Turin (Table 1).

The median CKD stage was 4, in both cohorts (Table 1). There was, however, a significant difference in the prevalence of obesity in the two cohorts (Le Mans 41.3% vs. Turin 15.0%, *p* < 0.001). Body mass index (BMI) was also significantly higher in Le Mans (median 29.0 kg/m^2^ compared to 25.4 kg/m^2^ in Turin, *p* < 0.001).

As a reflection of the different baseline dietary habits, protein intake at the time of quality of life evaluation was significantly higher in Le Mans than in Turin.

The median interval between the start of follow-up in the UIRAV, with the start of the diet, and the assessment of the QoL was 9 months.

It should be noted that the difference between the baseline protein intake (about 1.20 g/kg/day) and the protein intake at the time of the study was about 0.4 g/kg/day; while the pre-diet data was not available for the Italian control cohort, the median protein intake in this age group was estimated at about 1.0–1.1 kg/day in the area, thus at least partially accounting for the differences in protein intake when tested [31]. At the time of the start of care in the UIRAV the median albumin value was 3.7 g/dL (min 1.9–max 5.1) while at enrollment into the Pro-Re-Re-Pro study, nine months later, the median value was 3.8 g/dL (min 3.0–max 5.0), with a stable kidney function (median serum creatinine value at start was 2.17 mg/dl (min 0.94–max 8.70) vs. 2.18 (min 0.90–max 9.14) at enrollment into the Pro-Re-Re-Pro study)

### 3.2. Diet Distribution

At the time of the study, the cohort being followed in the UIRAV was almost evenly divided between normalization of protein intake, as a first step towards protein reduction, and a moderately restricted low-protein diet (Table 2).

The main clinical characteristics of the patients who started normalized or protein-restricted diets were significantly different: patients on a normal protein diet were older and had a higher comorbidity burden, while younger patients tended to be on a classic low-protein diet. Likewise, patients with lower e-GFR were more often on an LPD than on a normalized schedule. In this heterogeneous population, no difference was found on the Charlson Comorbidity Index at test.

Accordingly, no significant difference was found in the survival of patients according to the diet followed once they had been recruited in the study; the same holds true for dialysis start. Of note, patients on a normalized protein intake, associated with ketoacid and aminoacid supplementation, were older; they had higher comorbidity, a lower risk of dialysis start and a higher death rate, while the opposite is true for younger patients on supplemented LPDs. The resultant “total drop out” curve, in which the outcome was start of dialysis or death, does not show differences according to the diet at test (Supplementary Materials Figure S1a–c).

### 3.3. Quality of Life

There was no substantial difference in the reported quality of life according to different types of diet (normalization of the protein intake versus reduction in protein intake, with or without dietary supplements) at time of study. Older age (dichotomized at 70) and higher comorbidity (i.e., CCI ≥7) were associated with perception of poorer physical health perception, independently of diet (Table 3 and Table 4). Conversely, patients with worse kidney function were less satisfied with their social life (Table 3 and Table 4), again independently of the diet they were on.

In the multiple regression analysis, the odds of having a low QoL were not associated with any specific health determinant. Of note, the type of diet was not correlated with the risk of low QoL in any domain of QoL (Table 5).

### 3.4. Comparison between Settings

The comparison between the QoL scores in Le Mans and Turin confirms the lack of a significant effect of type of diet on patients’ quality of life in both settings (Supplementary Table S1).

However, the setting is associated with some minor differences in results, and notably the only domain showing a significant difference is the second one (social relationships). The most important difference was observed in patients’ satisfaction with their physical appearance, remarkably lower in the Italian than in the French cohort (Supplementary Materials Figure S2a–d).

### 3.5. Discussion

The fear of worsening the already impaired quality of life of patients with advanced CKD is one of the numerous barriers to the use of low-protein diets, in particular in elderly and high-comorbidity populations [1].

While there are many studies analyzing the effect of these diets on chronic kidney disease progression and metabolic interferences, as well as on mortality, there are comparatively few studies specifically addressed to LPDs’ potential effect on QoL.

The only large study is a multicenter analysis carried out in Italy, where the dietary approach to CKD has a longstanding tradition. Based on traditional Mediterranean foods, CKD care in Italy favors the reduction in protein intake without traumatic changes to dietary habits. Protein-free products are available and fully reimbursed by the National Health System [23]. The four participating centers shared a multiple-choice low-protein diet approach. In this context, patients’ quality of life correlated closely with the baseline conditions, in particular age and comorbidity, rather than with type and entity of dietary restriction. The presence of differences between centers indicated the possibility of a relevant modulation of QoL by cultural background and dietary habits.

Seen from this perspective, the present study sheds further light on this issue. The unit dedicated to the care of advanced CKD in Le Mans is representative of a different setting of study, i.e., a French rural area, in which patients are used to high-protein diets, as witnessed by a baseline protein intake of 1.2 g/kg/day.

Interestingly, the units in both Le Mans and Turin were set up by the same senior nephrologist, thus allowing a comparison of results within a similar approach to tailored dietary interventions, adapted to different situations.

As previously described, the original multiple-choice diet system developed in Italy was modified to account for the dietary habits in Central France, choosing a stepwise approach, starting from normalization of protein intake, followed by progressive protein restriction, with or without keto-analogue supplementation. Patients were encouraged to discuss the strategy best suited to reaching their target with the center’s dieticians [10,14]. Given that this was the approach adopted, it made it possible to evaluate the effect on QoL of being exposed to this flexible dietary “system” rather than the effect of different diets. The French cohort was comparable to the Italian one in terms of age and gender, reflecting the overall characteristics of the population with advanced CKD in Europe. However, the prevalence of obesity was significantly higher in Le Mans, as was the Charlson Comorbidity Index (Table 1). According to the REPOSI study, older age and male gender have been associated with mortality in hospitalized patients [32,33]. In this respect, our data, derived from an elderly cohort with a prevalence of men, underline the importance of a comprehensive pharmacological and nutritional approach in order to reduce mortality in advanced CKD patients starting form outpatient settings. Furthermore, in our study, about 40% of the patients were diabetic and the prevalence of proteinuria ≥1g/24 h was likewise high (Table 1 and Table 2). While diabetes *per se* and the presence of proteinuria have been associated with mortality and cardiovascular disease, the entity of proteinuria modulates the progression of CKD independently from diabetes [34]. Since moderately restricted LPDs are effective in both diabetic and non-diabetic patients, timely nutritional intervention can reduce the risk of CKD progression and related comorbidities [35]. As expected, given the known different baseline dietary habits, the protein intake at study was higher in the French cohort; however, the reduction in protein intake was also significant (about 0.4 g/kg/day from the start of combined nephrological and dietary management), and adherence was good enough to allow many patients to further reduce their protein intake over time (Table 2). A flexible attitude was also applied to the progressive changes in the diet allowing the patients to nearly or fully reach the target set; the number of diet consultations was, likewise, personalized. The median interval between the start of follow-up in the UIRAV and enrolment in the study was 9 months, which indeed corresponds to an initial adaptation time to the dietary changes prescribed.

In spite of the presence of advanced CKD, and of a high comorbidity burden, only a minority of the patients in the two settings rated their QoL as low, in each domain of the questionnaire. While physical health was described as poor by about 40% of the patients, psychological health and environment were rated as poor by only 9 to 22% of the patients. In this context, no difference was associated with the diet followed at QoL assessment (Table 4, Table 5 and Table 6).

The main difference in QoL in the two settings is in the social domain, and about half of this difference is in answers to the question regarding physical appearance, considered either acceptable or “not relevant” by the French cohort and “impaired” by the Italian one.

Overall, our data are in line with those recorded in elderly patients in France and Italy [36,37,38,39]. These findings, together with the lack of influence of the diet followed on the QoL, show that low-protein diets can be safely used even for elderly and high comorbidity populations, and in settings where they have not routinely been prescribed.

Our study has several limitations: it lacks a control group with a similar level of CKD but not on a low-protein diet. This could not be avoided, given that the diet is systematically offered to all patients, and the few that refuse to at least try it and see if the results are beneficial can be expected to be clinically and psychologically different as compared with those who adhere to the program [14]. Furthermore, although the data were all gathered within a reasonably short period of time, and in settings with a similar philosophy of care, the dietary evaluation was performed by different dieticians on the basis of their experience, and the study exploits a historic cohort for comparison [10]. Finally, we did not assess the carbohydrate or fat intake at baseline in our population, which could be significant determinants of obesity and diabetes. However, the baseline dietary pattern of our population are unlikely to differ from the one described in the INCA2 survey, showing an average intake for total fat of 75.5 and 205.6 g/day for carbohydrates [38]. The strengths of the study, exploring a relatively poorly known aspect of the dietary management of CKD patients, are in the relatively large on-diet cohort enrolled and the comparison of two countries in which a similar system of care was set up.

Our results show that reducing protein intake is feasible in different settings, even for patients who, because of age and comorbidity, are often considered poor candidates for dietary management. While we believe that a flexible approach, respecting patients’ preferences, as much as possible, played a major role in acceptance and perceived low intrusiveness of the diets prescribed; further comparisons with other settings and approaches on a larger scale are much needed to highlight differences and help identify the best strategies to adopt to expand the use of these important and probably underexploited dietary tools in CKD care.

## 4. Conclusions

In conclusion, our study, performed in a rural setting in Central France, exploring the QoL of patients with advanced CKD treated in a dedicated unit, suggests that a protein restricted diet is not associated with impairment of quality of life, and indirectly supports a personalized, stepwise approach to prescribing low-protein diets. Further multicentric studies are needed to corroborate this result in different populations with different dietary habits on a larger scale, to facilitate the implementation of the favorable results described in randomized trials and summarized in the current guidelines.

## Figures and Tables

**Table 1:** Baseline characteristics of the population, in the UIRAV unit, and in the Turin unit.

	UIRAV—Le Mans	Turin—S. Luigi	*p*-Values
*N*	153	128	
Age (years), median (min-max)	73 (23–96)	74 (20–90)	0.217
Gender, *n* (%)			0.619
Male	100 (65.4%)	80 (62.5%)	
Female	53 (34.6%)	48 (37.5%)	
BMI (kg/m^2^), median (min-max)	29.0 (18.6–51.2)	25.4 (17.3–39.5)	**<0.001**
CCI, median (min-max)	7 (2–17)	6 (2–11)	**0.023**
MIS, median (min-max)	5 (1–12)	-	-
SGA, *n* (%)		-	-
A	139 (90.8%)		
B	14 (9.2%)		
C	0		
Diabetes, *n* (%)	67 (43.8%)	46 (35.9%)	0.181
Obesity (>30 kg/m^2^), *n* (%)	63 (41.2%)	19 (15.0%)	**<0.001**
Kidney diseases, *n* (%)			**<0.001**
Glomerulonephritis + systemic	4 (2.6%)	21 (16.4%)	
Nephroangiosclerosis + diabetic	106 (69.3%)	49 (38.3%)	
ADPKD	6 (3.9%)	13 (10.2%)	
Others	37 (24.2%)	35 (27.3%)	
Proteinuria (g/day), median (min-max)	0.37 (0.03–10.24)	0.93 (0.01–9.65)	0.139
Proteinuria ≥ 3 g/day, *n* (%)	24 (15.7%)	13 (10.2%)	0.172
Creatinine, (mg/dL), median (min-max)	2.17 (0.94–8.70)	3.04 (0.66–15.66)	**<0.001**
eGFR-CKD EPI (mL/min/1.73 m^2^), median(min-max)	27 (5–58)	18 (3–73)	**<0.001**
CKD stages, *n* (%)			**<0.001**
2	0	4 (2.6%) *	
3A	8 (5.2%)	6 (3.9%)	
3B	54 (35.3%)	15 (11.7%)	
4	74 (48.4%)	56 (43.8%)	
5	17 (11.1%)	47 (36.7%)	
Protein intake at Pro-Re-Re-Pro (g/kg/day), median (min-max)	0.80 (0.40–1.40)	0.50 (0.31–1.03)	**<0.001**
Protein intake at UIRAV (g/kg/day), median (min-max)	1.20 (0.65–1.70)	-	

CKD, chronic kidney disease; BMI, body mass index; CCI, Charlson Comorbidity Index; HbA1c, glycated hemoglobin; PTH, parathyroid hormone; ADPKD, autosomal dominant polycystic kidney disease; eGFR, glomerular filtration rate estimated by the CKD-EPI equation. * These patients started the diet in CKD stage 3 and continued it after improvement. Bolds highlight the significant differences.

**Table 2:** Diet distribution in the UIRAV patients.

	Normalization of Protein Intake (0.8 g/kg/day)		Low-Protein Diets (≥0.6 g/kg/day)		All	*p*-Values
	Non-Supplemented	Supplemented	Non-Supplemented	Supplemented		Among Diets
*N*	57	8	59	29	153	
Age (years), median (min–max)	77 (23–96)	85 (61–94)	72 (37–96)	66 (33–94)	73 (23–96)	**0.029**
Age ≥ 65 years, *n* (%)	46 (80.7%)	6 (75%)	40 (67.8%)	17 (58.6%)	109 (71.2%)	0.162
Age ≥ 80 years, *n* (%)	23 (40.4%)	5 (62.5%)	12 (20.3%)	6 (20.7%)	46 (30.1%)	**0.013**
Gender, *n* (%)						0.136
Male	37 (64.9%)	6 (75%)	43 (72.9%)	14 (48.3%)	100 (65.4%)	
Female	20 (35.1%)	2 (25%)	16 (27.1%)	15 (51.7%)	53 (34.6%)	
BMI (kg/m^2^), median (min–max)	28.7 (19.5 (42.7)	25.3 (18.6–50.0)	29.6 (19.0–51.2)	29.1 (21.6–41.9)	29 (18.6–51.2)	0.198
CCI, median (min–max)	7 (2–13)	8 (5–17)	7 (2–10)	7 (2–10)	7 (2–17)	0.257
CCI ≥ 7, *n* (%)	38 (66.7%)	7 (87.5%)	39 (66.1%)	16 (55.2%)	100 (65.4%)	0.374
CCI ≥ 10, *n* (%)	10 (17.5%)	3 (37.5%)	5 (8.5%)	2 (6.9%)	20 (13.1%)	0.064
Diabetes, *n* (%)	16 (28.1%)	2 (25.0%)	30 (50.8%)	19 (65.5%)	67 (43.8%)	**0.029**
HbA1c%, median (min–max)	5.86 (4.75–11.18)	5.81 (4.59–6.76)	6.15 (5.30–9.71)	6.28 (4.71–9.54)	6.01 (4.59–11.18)	0.098
PTH, median (min-max)	56 (8–281)	70 (25–408)	105 (2–986)	104 (30–962)	76 (2–986)	**0.003**
Neoplasia, *n* (%)	11 (19.3%)	2 (25.0%)	6 (10.2%)	4 (13.8%)	23 (15.0%)	0.384
Obesity, *n* (%)	19 (33.3%)	3 (37.5%)	28 (47.5%)	13 (44.8%)	63 (41.2%)	0.453
ADPKD, *n* (%)	2 (3.5%)	0	2 (3.4%)	2 (6.9%)	6 (3.9%)	0.817
Glomerulonephritis-systemic disease, *n* (%)	2 (3.5%)	1 (12.5%)	1 (1.7%)	0	4 (2.6%)	0.244
Creatinine, (mg/dL), median (min–max)	1.90 (0.94–7.05)	2.20 (1.43–3.80)	2.32 (1.46–5.72)	2.58 (1.14–8.71)	2.17 (0.94–8.71)	**0.001**
eGFR-EPI (mL/min/1.73 m^2^), median (min–max) Ɨ	31 (5–58)	30 (10–40)	25 (9–39)	20 (6–45)	27 (5–58)	**<0.001**
CKD stage, *n* (%)						**0.008**
3 A	7 (12.3%)	0	0	1 (3.5%)	8 (5.2%)	
3 B	26 (45.6%)	4 (50.0%)	19 (32.2%)	5 (17.2%)	54 (35.3%)	
4	19 (33.3%)	3 (37.5%)	35 (59.3%)	17 (58.6%)	74 (48.4%)	
5	5 (8.8%)	1 (12.5%)	5 (8.5%)	6 (20.7%)	17 (11.1%)	
Proteinuria (g/day), median (min–max)	0.24 (0.09–10.24)	1.70 (0.14–4.54)	0.43 (0.03–5.49)	1.44 (0.04–8.26)	0.37 (0.03–10.24)	**0.003**
Proteinuria ≥ 1 g/day, *n* (%)	12 (24.5%)	5 (71.4%)	18 (36%)	18 (62.1%)	53 (39.3%)	**0.003**
Proteinuria ≥ 3 g/day, *n* (%)	9 (18.4%)	2 (28.6%)	4 (8%)	9 (31%)	24 (15.7%)	0.062
Protein intake at Pro-Re-Re-Pro study (g/kg/day), median (min–max)	1.00 (0.50–1.40)	1.00 (0.80–1.20)	0.70 (0.40–1.20)	0.60 (0.40–1.00)	0.80 (0.40–1.40)	**<0.001**
Protein intake pre-UIRAV, (g/kg/day), median (min–max)	1.20 (0.70–1.70)	1.15 (0.90–1.40)	1.10 (0.65–1.50)	1.20 (0.80–1.50)	1.20 (0.65–1.70)	0.132
Follow-up at Pro-Re-Re-Pro (months), median (min–max)	7 (0–26)	7 (2–19)	9 (0–18)	14 (1–19)	9 (0–26)	**0.002**

Bolds highlight the significant differences.

**Table 3:** Quality of life in patients sorted according to their diet prescriptions.

	Normalization of Protein Intake (0.8 g/kg/day)		Low Protein Diets (≥0.6 g/kg/day)		A	
	Non-Supplemented	Supplemented	Non-Supplemented	Supplemented		*p*-Values Among Diets
*N*						
Quality of life, median (min–max)						
Physical health	3.21 (2.00–4.71)	3.33 (2.83–4.00)	3.33 (2.00–4.17)	3.33 (2.29–4.33)	3.31 (2.00–4.71)	0.547
Psychological health	4.00 (2.40–5.00)	4.00 (3.50–4.50)	3.92 (2.00–5.00)	4.17 (3.00–4.67)	4.00 (2.00–5.00)	0.679
Social relationships	3.83 (1.00–5.00)	3.33 (2.67–4.33)	4.00 (1.67–5.00)	3.67 (2.00–5.00)	3.67 (1.00–5.00)	0.513
Environment	3.63 (2.63–4.75)	4.00 (3.25–4.75)	3.81 (2.38–4.75)	3.88 (2.25–4.63)	3.75 (2.25–4.75)	0.746

Bolds highlight the significant differences.

**Table 4:** Relationship between the main health determinants, diet and QoL.

	Physical Health	*n* = 140			Psychological Health	*n* = 140		
	Poor	Average	Good	*p*-Value	Poor	Average	Good	*p*-Value
	*n* (Row %)	*n* (Row %)	*n* (Row %)		*n* (Row %)	*n* (Row %)	*n* (Row %)	
*N*	47	75	18		7	59	75	
Diet, *n* (%)				0.593				0.197
Normal intake 0.8 g/kg/day	22 (41.5%)	24 (45.3%)	6 (11.3%)		2 (3.8%)	24 (45.3%)	27 (50.9%)	
Supplemented 0.8 g/kg/day	1 (14.3%)	5 (71.4%)	1 (14.3%)		0 (0%)	3 (42.9%)	4 (57.1%)	
LPD 0.6 g/kg/day	7 (25.9%)	15 (55.6%)	5 (18.5%)		0 (0%)	10 (37%)	17 (63%)	
LPD Supplemented 0.6 g/kg/day	17 (31.5%)	31 (57.4%)	6 (11.1%)		5 (9.3%)	22 (40.7%)	27 (50%)	
eGFR mL/min/1.73 m^2^, *n* (%)				0.999				0.214
≥15	42 (33.1%)	68 (53.5%)	16 (12.6%)		5 (3.9%)	53 (41.7%)	69 (54.3%)	
<15	5 (35.7%)	7 (50%)	2 (14.3%)		2 (14.3%)	6 (42.9%)	6 (42.9%)	
Gender, *n* (%)				0.1783				**0.007**
Male	28 (29.2%)	56 (58.3%)	11 (11.5%)		3 (3.1%)	48 (50%)	45 (46.9%)	
Female	19 (42.2%)	19 (42.2%)	7 (15.6%)		4 (8.9%)	11 (24.4%)	30 (66.7%)	
Age (years), *n* (%)				**0.018**				0.459
≥70	35 (42.7%)	37 (45.1%)	9 (11%)		5 (6.1%)	37 (45.1%)	40 (48.8%)	
<70	12 (20.3%)	38 (64.4%)	9 (15.3%)		2 (3.4%)	22 (37.3%)	35 (59.3%)	
CCI, *n* (%)				**0.015**				0.089
<7	9 (18%)	33 (66%)	8 (16%)		3 (6%)	15 (30%)	32 (64%)	
≥7	38 (41.8%)	42 (46.2%)	10 (11%)		4 (4.4%)	44 (48.4%)	43 (47.3%)	
	**Social relationships**				**Environment**			
*N*	13	57	69		13	91	37	
Diet, n (%)				0.696				0.884
Normal intake 0.8 g/kg/day	5 (9.4%)	21 (39.6%)	26 (49.1%)		6 (11.3%)	35 (66%)	12 (22.6%)	
Supplemented 0.8 g/kg/day	1 (14.3%)	4 (57.1%)	2 (28.6%)		0 (0%)	4 (57.1%)	3 (42.9%)	
LPD 0.6 g/kg/day	1 (3.7%)	13 (48.1%)	12 (44.4%)		3 (11.1%)	18 (66.7%)	6 (22.2%)	
LPD Supplemented 0.6 g/kg/day	6 (11.1%)	19 (35.2%)	29 (53.7%)		4 (7.4%)	34 (63%)	16 (29.6%)	
eGFR mL/min/1.73m^2^, *n* (%)				0.371				0.128
≥15	11 (8.7%)	50 (39.4%)	64 (50.4%)		11 (8.7%)	85 (66.9%)	31 (24.4%)	
<15	2 (14.3%)	7 (50%)	5 (35.7%)		2 (14.3%)	6 (42.9%)	6 (42.9%)	
Gender, *n* (%)				0.549				0.268
Male	10 (10.4%)	41 (42.7%)	44 (45.8%)		11 (11.5%)	58 (60.4%)	27 (28.1%)	
Female	3 (6.7%)	16 (35.6%)	25 (55.6%)		2 (4.4%)	33 (73.3%)	10 (22.2%)	
Age (years), *n* (%)				0.212				0.484
≥70	10 (12.2%)	29 (35.4%)	41 (50%)		6 (7.3%)	56 (68.3%)	20 (24.4%)	
<70	3 (5.1%)	28 (47.5%)	28 (47.5%)		7 (11.9%)	35 (59.3%)	17 (28.8%)	
CCI, *n* (%)				0.096				0.963
<7	3 (6%)	16 (32%)	31 (62%)		4 (8%)	33 (66%)	13 (26%)	
≥7	10 (11%)	41 (45.1%)	38 (41.8%)		9 (9.9%)	58 (63.7%)	24 (26.4%)	

Bolds highlight the significant differences.

**Table 5:** Multiple logistical regression for the outcome poor quality of life in the UIRAV cohort.

	Odds-Ratio	Lower	Higher	*p*-Values
**QoL Domain 1 (Physical health)**				
Age (≥70 years old)	1.698	0.667	4.326	0.267
Gender (Males vs. Females)	0.515	0.234	1.134	0.099
eGFR (<20 mL/min)	0.709	0.292	1.725	0.449
CCI (≥7)	2.578	0.943	7.045	0.065
0.6 vs. 0.8 g/kg/day of protein intake	0.750	0.352	1.598	0.456
**QoL domain 2 (Psychological health)**				
Age (≥70 years old)	4.705	0.467	47.417	0.189
Gender (Males vs. Females)	0.333	0.069	1.614	0.172
eGFR (<20 mL/min)	1.198	0.196	7.320	0.845
CCI (≥7)	0.314	0.040	2.444	0.269
0.6 vs. 0.8 g/kg/day of protein intake	2.110	0.372	11.974	0.399
**QoL domain 3 (Social Relationships)**				
Age (≥70 years old)	2.590	0.524	12.791	0.243
Gender (Males vs. Females)	1.690	0.435	6.572	0.449
eGFR (<20 mL/min)	1.491	0.412	5.400	0.543
CCI (≥7)	1.118	0.226	5.525	0.891
0.6 vs. 0.8 g/kg/day of protein intake	0.915	0.282	2.972	0.882
**QoL domain 4 (Environment)**				
Age (≥70 years old)	0.425	0.111	1.626	0.211
Gender (Males vs. Females)	2.480	0.513	11.984	0.259
eGFR (<20 mL/min)	0.911	0.226	3.673	0.896
CCI (≥7)	1.890	0.451	7.919	0.384
0.6 vs. 0.8 g/kg/day of protein intake	0.811	0.246	2.674	0.731

**Table 6:** Multiple logistical regression for the outcome poor quality of life in Le Mans and Turin.

	Odds-Ratio	Lower	Higher	*p*-Values
**QoL Domain 1 (Physical Health)**				
Age (≥70 years old)	1.88	0.987	3.581	0.055
Gender (Males vs. Females)	0.361	0.199	0.654	**0.001**
eGFR (<20 mL/min)	1.082	0.59	1.986	0.799
CCI (≥7)	3.673	1.867	7.228	**<0.001**
Setting (Turin vs. Le Mans)	0.82	0.446	1.508	0.524
**QoL domain 2 (Psychological)**				
Age (≥70 years old)	1.525	0.722	3.22	0.268
Gender (Males vs. Females)	0.561	0.278	1.133	0.107
eGFR (<20 mL/min)	0.869	0.428	1.765	0.697
CCI (≥7)	1.536	0.738	3.199	0.251
Setting (Turin vs. Le Mans) *	10.168	4.107	25.176	**<0.001**
**QoL domain 3 (Social relationships)**				
Age (≥70 years old)	1.116	0.49	2.541	0.793
Gender (Males vs. Females)	1.958	0.83	4.615	0.125
eGFR (<20 mL/min)	2.141	0.98	4.676	0.056
CCI (≥7)	1.62	0.702	3.741	0.258
Setting (Turin vs. Le Mans)	1.706	0.764	3.811	0.193
**QoL domain 4 (Environment)**				
Age (≥70 years old)	0.266	0.101	0.697	**0.007**
Gender (Males vs. Females)	0.933	0.368	2.364	0.883
eGFR (<20 mL/min)	1.257	0.499	3.169	0.628
CCI (≥7)	3.364	1.204	9.401	**0.021**
Setting (Turin vs. Le Mans)	0.867	0.343	2.19	0.763

* Question no. 11 «Do you accept your physical appearance?», shows a different levels of acceptance of physical appearance by patients in the Le Mans and Turin cohorts, as shown in Supplementary Figure S2. The odds ratio of the setting without this question is 5.598 (2.089–15.003) for *p* < 0.001. Bolds highlight the significant differences.

## Data Availability

The data presented in this study are available on request from the corresponding author.

## Supplementary Materials

The following are available online at https://www.mdpi.com/article/10.3390/nu13041354/s1, Table S1: Multiple logistical regression for the outcome poor quality of life in the Turin cohort, Figure S1: Survival analysis (Kaplan-Meier) for the outcome: death, dialysis and death or dialysis, Figure S2: QoL answers in different domains: comparison between settings.
