# Supplementary material for: Quality of Life in CKD Patients on Low-Protein Diets in a Multiple-Choice Diet System. Comparison between a French and an Italian Experience
Source: Nutrients. 2021 Apr 18;13(4):1354. doi: 10.3390/nu13041354 (PMC8073895; doi:10.3390/nu13041354)
Supplement: Supplementary file 1 [file nutrients-13-01354-s001.zip › nutrients-1167031-supplementary.pdf]

SUPPLEMENTARY MATERIAL

Supplementary Table S1. Multiple logistical regression for the outcome poor quality of life in the Turin cohort

**QoL domain 1 (Physical health)**	Odds-ratio	Lower	Higher	p-values
Age (≥70 years old)	2.650	0.926	7.588	0.069
Gender (Males vs. Females)	0.189	0.069	0.518	**0.001**
eGFR (<20 ml/min)	1.801	0.699	4.639	0.223
CCI (≥7)	6.211	2.274	16.960	**<0.001**
0.6 vs. 0.8 g/kg/day of protein intake	1.027	0.538	1.961	0.936

**QoL domain 2 (Psychological health)**	Odds-ratio	Lower	Higher	p-values
Age (≥70 years old)	1.545	0.657	3.630	0.318
Gender (Males vs. Females)	0.614	0.275	1.375	0.236
eGFR (<20 ml/min)	0.788	0.356	1.747	0.558
CCI (≥7)	1.945	0.878	4.308	0.101
0.6 vs. 0.8 g/kg/day of protein intake	1.163	0.663	2.039	0.598

**QoL domain 3 (Social relationships)**	Odds-ratio	Lower	Higher	p-values
Age (≥70 years old)	0.459	0.150	1.403	0.172
Gender (Males vs. Females)	2.788	0.888	8.755	0.079
eGFR (<20 ml/min)	4.105	1.304	12.922	**0.016**
CCI (≥7)	1.456	0.511	4.147	0.482
0.6 vs. 0.8 g/kg/day of protein intake	0.374	0.148	0.940	**0.037**

**QoL domain 4 (Environment)**	Odds-ratio	Lower	Higher	p-values
Age (≥70 years old)	0.187	0.038	0.913	**0.038**
Gender (Males vs. Females)	0.394	0.096	1.608	0.194
eGFR (<20 ml/min)	2.283	0.488	10.681	0.294
CCI (≥7)	5.681	1.177	27.419	**0.031**
0.6 vs. 0.8 g/kg/day of protein intake	1.038	0.383	2.810	0.942

* Seven patients prescribed very low-protein diets (0.3 g/kg/day) were not considered for this analysis.

Supplementary Figure S1

a. Survival analysis (Kaplan-Meier) for the outcome: death

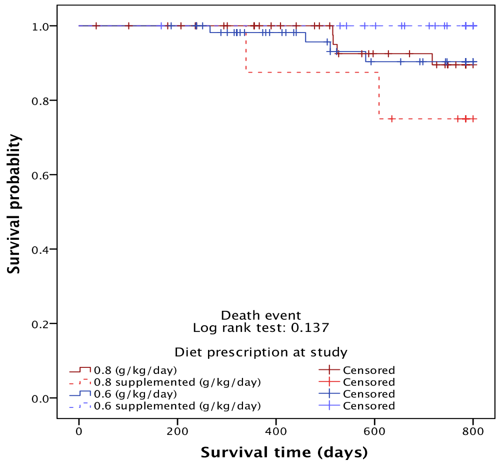

b. Survival analysis (Kaplan-Meier) for the outcome: dialysis

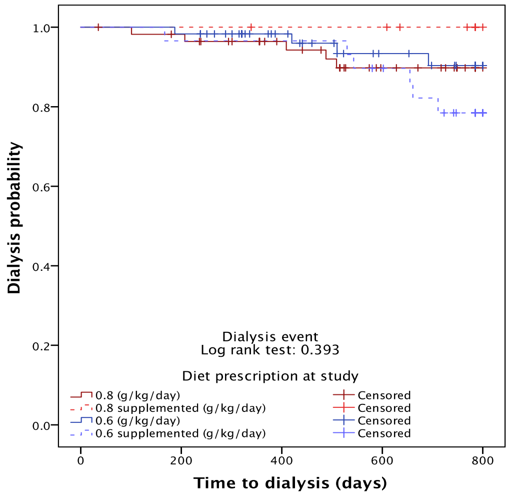

c. Survival analysis (Kaplan-Meier) for the outcome: death or dialysis

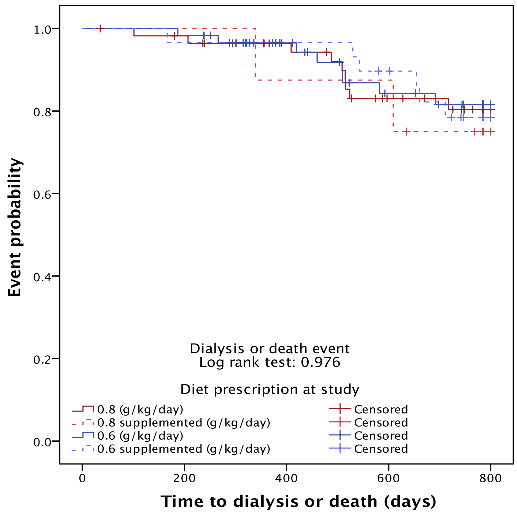

Supplementary Figure S2: QoL answers in different domains: comparison between settings (a)

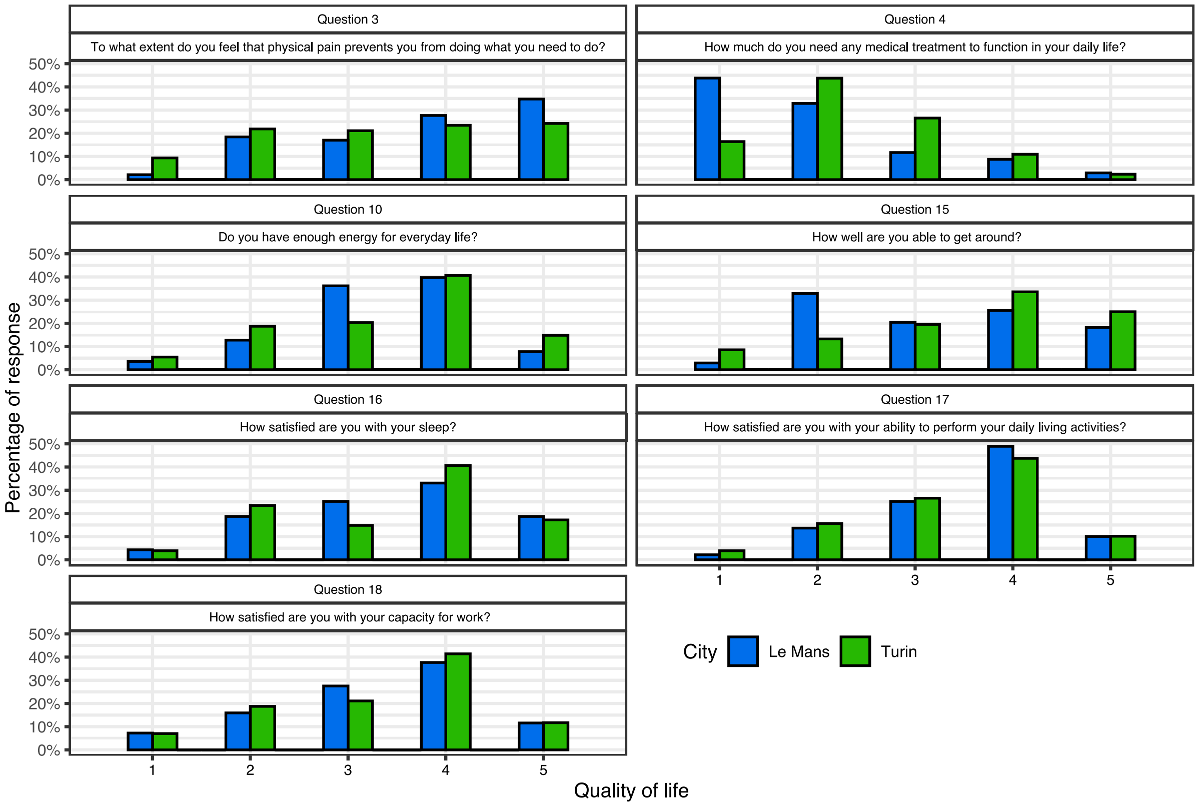

(b)

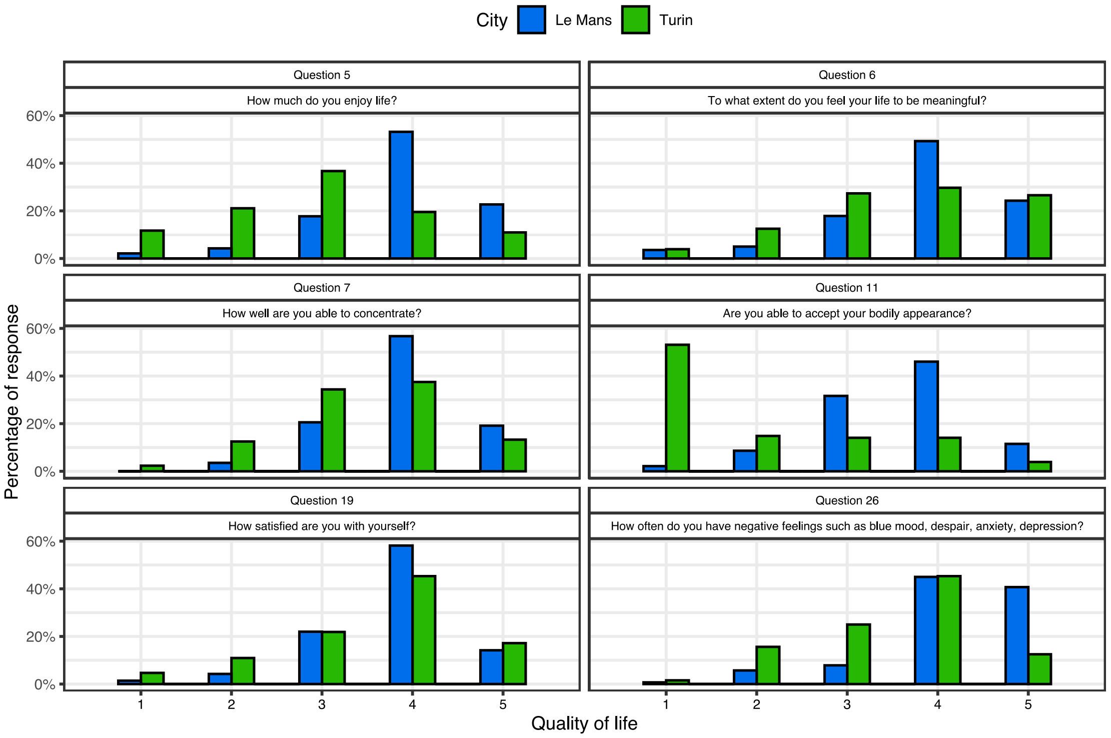

(c)

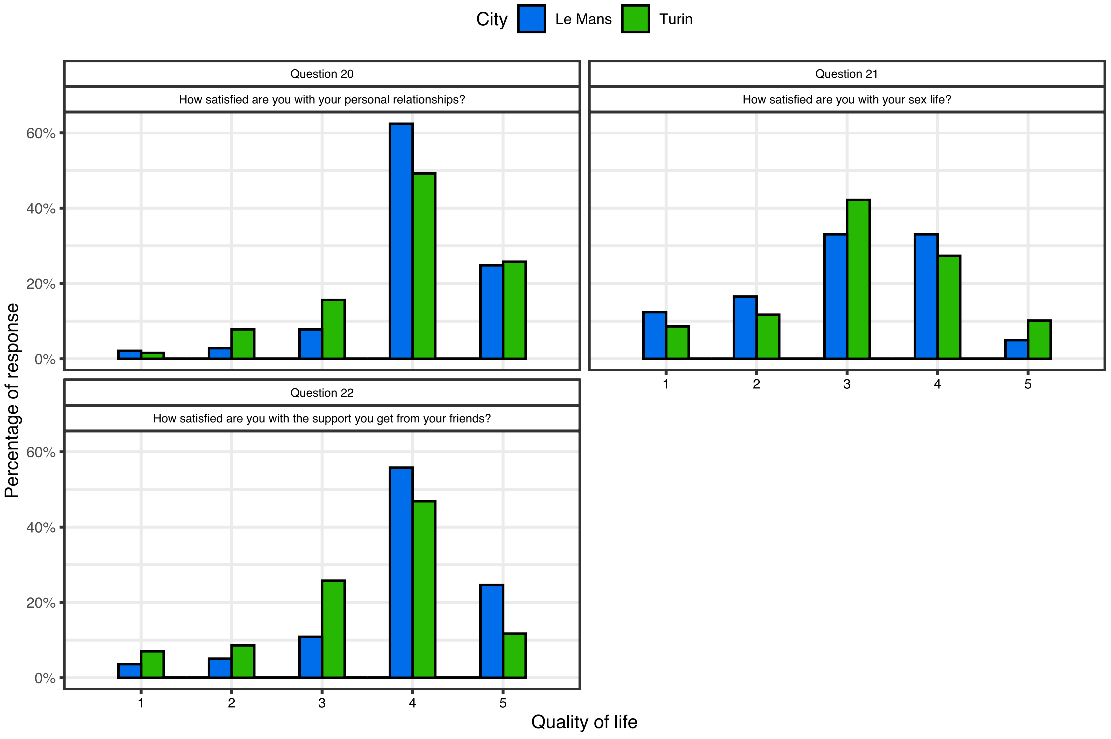
City
Le Mans
Turin
Question 20
How satisfied are you with your personal relationships?
Question 21
How satisfied are you with your sex life?
Question 22
How satisfied are you with the support you get from your friends?
60%
40%
20%
0%
Percentage of response
1
2
3
4
5
Quality of life

(d)

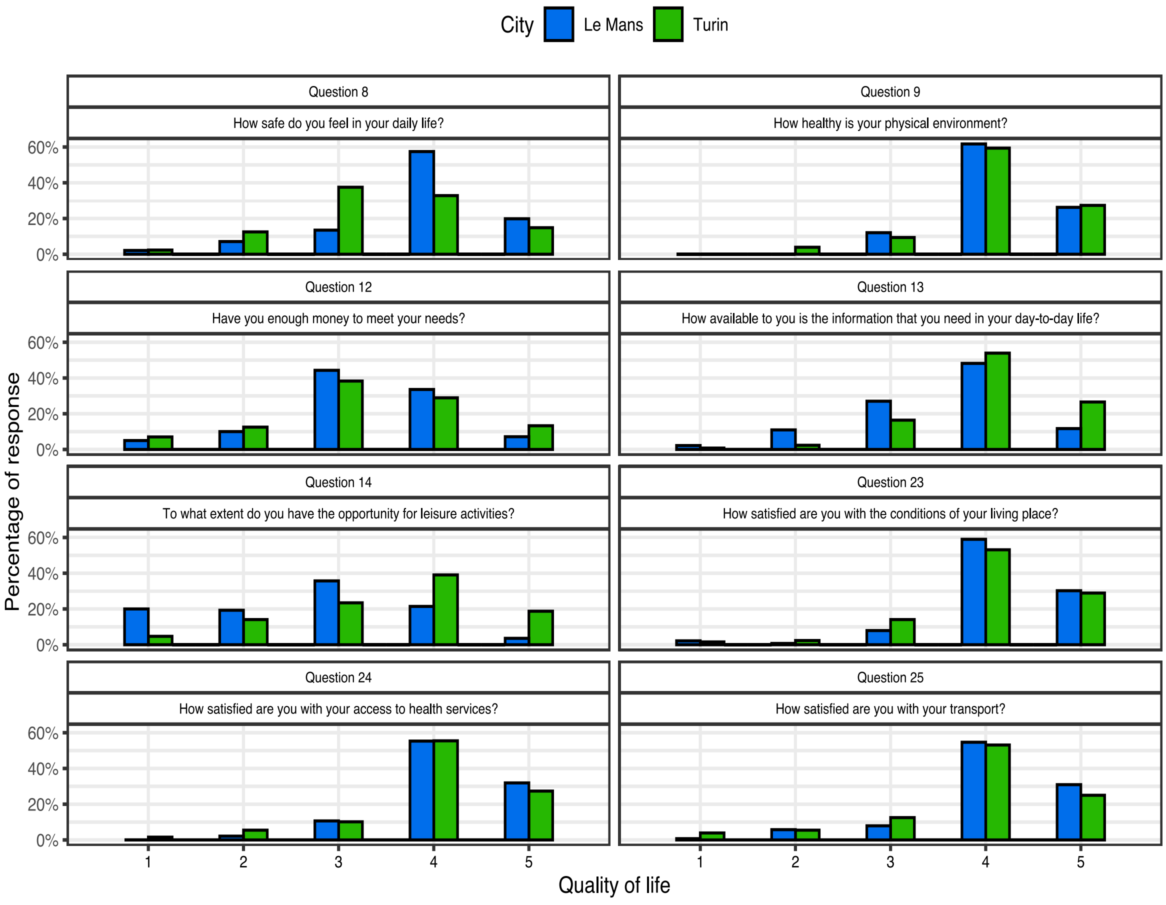
City
Le Mans
Turin
Question 8
How safe do you feel in your daily life?
Question 9
How healthy is your physical environment?
Question 12
Have you enough money to meet your needs?
Question 13
How available to you is the information that you need in your day-to-day life?
Question 14
To what extent do you have the opportunity for leisure activities?
Question 23
How satisfied are you with the conditions of your living place?
Question 24
How satisfied are you with your access to health services?
Question 25
How satisfied are you with your transport?
60%
40%
20%
0%
Percentage of response
1
2
3
4
5
Quality of life
